# Reproductive Potential of Yeast Cells Depends on Overall Action of Interconnected Changes in Central Carbon Metabolism, Cellular Biosynthetic Capacity, and Proteostasis

**DOI:** 10.3390/ijms21197313

**Published:** 2020-10-03

**Authors:** Roman Maslanka, Renata Zadrag-Tecza

**Affiliations:** Department of Biochemistry and Cell Biology, Institute of Biology and Biotechnology, College of Natural Sciences, University of Rzeszow, 35-601 Rzeszow, Poland; romekmaslanka@gmail.com

**Keywords:** hexokinase, cell size, reproductive potential, biosynthetic capacity, proteostasis, calorie restriction

## Abstract

Carbon metabolism is a crucial aspect of cell life. Glucose, as the primary source of energy and carbon skeleton, determines the type of cell metabolism and biosynthetic capabilities, which, through the regulation of cell size, may affect the reproductive capacity of the yeast cell. Calorie restriction is considered as the most effective way to improve cellular physiological capacity, and its molecular mechanisms are complex and include several nutrient signaling pathways. It is widely assumed that the metabolic shift from fermentation to respiration is treated as a substantial driving force for the mechanism of calorie restriction and its influence on reproductive capabilities of cells. In this paper, we propose another approach to this issue based on analysis the connection between energy-producing and biomass formation pathways which are closed in the metabolic triangle, i.e., the respiration-glycolysis-pentose phosphate pathway. The analyses were based on the use of cells lacking hexokinase 2 (∆*hxk2*) and conditions of different glucose concentration corresponding to the calorie restriction and the calorie excess. Hexokinase 2 is the key enzyme involved in central carbon metabolism and is also treated as a calorie restriction mimetic. The experimental model used allows us to explain both the role of increased respiration as an effect of calorie restriction but also other aspects of carbon metabolism and the related metabolic flux in regulation of reproductive potential of the cells. The obtained results reveal that increased respiration is not a prerequisite for reproductive potential extension but rather an accompanying effect of the positive role of calorie restriction. More important seems to be the changes connected with fluxes in central carbon metabolic pathways resulting in low biosynthetic capabilities and improved proteostasis.

## 1. Introduction

Cell growth and reproduction are important cell properties in unicellular and multicellular organisms alike. In the case of yeast cells, the ability to reproduce, or reproductive potential, is expressed by the number of daughter cells produced by the mother cell during her lifetime [1]. The reproductive potential of yeast cells is limited, and both genetic and environmental factors have an impact on this parameter. For instance, mutations in numerous genes increase or decrease the numerical value of reproductive potential [2,3,4]; also, changes in nutrient content in the medium lead to substantial increase (e.g., in the calorie restriction condition) [5,6] or decrease (e.g., in the conditions of glucose excess) [7] of that potential. Cell size and the rate of cell size increase per generation also play an important role in the regulation of reproductive potential [8,9,10,11]. More importantly, those parameters may be modulated, among others, by nutrient content [12,13].

Carbon metabolism is a crucial aspect of cell life entailing a large number of consequences, including impact on the reproductive capacity of cells. One of the best-known examples of such aspects is the phenomenon of calorie restriction (CR), which is the most effective way of extending life observed in a wide range of species [14,15]. In the case of yeast cells, CR conditions are achieved by reducing glucose concentration, usually from 2% to 0.5% [16]. The proposed molecular mechanisms of CR-mediated improvement of reproductive capacity of cells are complex and include several nutrient signaling pathways, among which are the Ras/cAMP/PKA signaling pathway [16,17,18] or TOR (Target of Rapamycin) and the serine/threonine protein kinase Sch9 [19]. Moreover, the metabolic shift from fermentation to mitochondrial respiration is treated as a substantial driving force behind the mechanism of CR action and its influence on yeast lifespan. It was proposed that increase in respiration leads to increase in the NAD^+^/NADH ratio, which in turn activates the NAD-dependent histone deacetylase Sir2 required for lifespan extension in CR conditions [16,20]. Another mechanism of Sir2p activation suggested that by inhibition of the TOR pathway and the Msn2/4 transcription factors CR upregulates the stress response gene *PNC1*. In turn, the increased level of the nicotinamidase Pnc1 activates Sir2p by decreasing the levels of its inhibitor—nicotinamide [21,22]. However, there are also several reports postulating Sir2p-independent action of CR [23,24]. Equally intriguing are the data indicating that CR increases lifespan also in respiratory-deficient yeast cells (rho^0^), which shows that some aspects of CR appear to be independent of respiration [25,26].

Cellular carbon metabolism is especially associated with glucose, which is the basic energy substrate. Yeast cells can use three types of glucose metabolism, from fermentation preferred in high glucose conditions through respire-fermentative metabolism to respiration where glucose content in a cell growth environment is reduced [27,28,29]. The *S. cerevisiae* yeast cells prefer alcoholic fermentation until the glucose reaches a low level. This phenomenon known as the Crabtree effect has not yet been fully understood [30,31]; therefore, the current studies postulate explanations connected with existence of specific cellular economics [32]. Glucose is not only the preferential substrate for energy yielding metabolism but also may act as a signaling molecule. It provides also the carbon skeleton used for biosynthesis of important cell macromolecules, such as nucleotides, amino acids, lipids, and cofactors of enzymatic reactions [33], which is particularly important during cell proliferation. The growth of the cell and its proliferation are highly energy-consuming processes, yet cells must be able to adapt their metabolism to nutrients availability, which is the reason for a specific cellular metabolic trade-off related with specific costs and benefits of different types of metabolism [32,34].

Glucose metabolism is quite well understood. However, there are still many questions concerning the so-called metabolic flux and the mechanism of cooperation between pathways composing the central carbon metabolism. Among them, especially important are questions concerning dependencies between glucose metabolism and ability of cells to reproduce both in calorie restriction and, of particular importance, in calorie excess conditions. The analyses were based on the use of the Δ*hxk2* strain (a strain lacking hexokinase 2 (Hxk2p)), and the calorie restriction and calorie excess conditions. Hxk2p is one of the three hexokinase isoenzymes present in the *S. cerevisiae*; the other two are hexokinase 1 (Hxk1p) and glucokinase (Glk1p). During growth on glucose Hxk2p is the main isoenzyme which, besides acting as a glycolytic enzyme, is involved in central carbon metabolism (CCM). Together with Mig1p, Mig2p, Reg1p, and Snf1p, Hxk2p formed a functional repressor complex which, binding to the *SUC2* promoter, repressed expression of several genes, such as genes encoding high-affinity glucose transporters, as well as genes responsible for mitochondrial activity and respiratory metabolism [29,35,36]. Moreover, in response to high glucose level in the medium, Hxk2p also repressed the expression of the *HXK1* and *GLK1* genes [37]. Therefore, hexokinase 2 plays an important role in the intracellular glucose-sensing process [29,36]. The aim of the study was to explain the links between glucose metabolism and reproductive capacity of the yeast cells. The experimental model used made it possible to explain not only the role of increased respiration as an effect of CR but also other aspects of carbon metabolism and the related metabolic flux in regulation of reproductive potential of the cells. For those purposes, we assessed the reproductive potential, cellular biosynthetic capacity, proteasomal activity, and parameters connected with the carbon metabolism pathways for the cells growing in the condition of different glucose concentrations. Those parameters are related to the specific metabolic triangle: the respiration—glycolysis—pentose phosphate pathway. The obtained results showed that, besides increasing respiration, deletion of the *HXK2* gene results in redirection in glucose-utilization pathway. This entails a decrease in cellular biosynthetic capabilities, improved proteostasis, and an increase in reproductive potential of the cells. These results suggest that the beneficial effect of the calorie restriction on reproductive capabilities of the cells is not the result of increased respiration per se, but it is rather effect of the overall action of interconnected changes in CCM.

## 2. Results

### 2.1. Lack of Hexokinase 2 Prevents Decrease in Reproductive Potential Caused by Increasing Glucose Concentration

The carbon/metabolic flux is a crucial response mechanism to changes in nutrient availability, which is directly connected with cell reproductive capacity by influencing physiological efficiency of cells. As we previously reported, although calorie excess significantly decreases reproductive potential and total lifespan of yeast cells, the decreased activity of cAMP-PKA pathway (deletion of Gpa2p and Gpr1p) reduces these changes [7].

**Figure 1:**
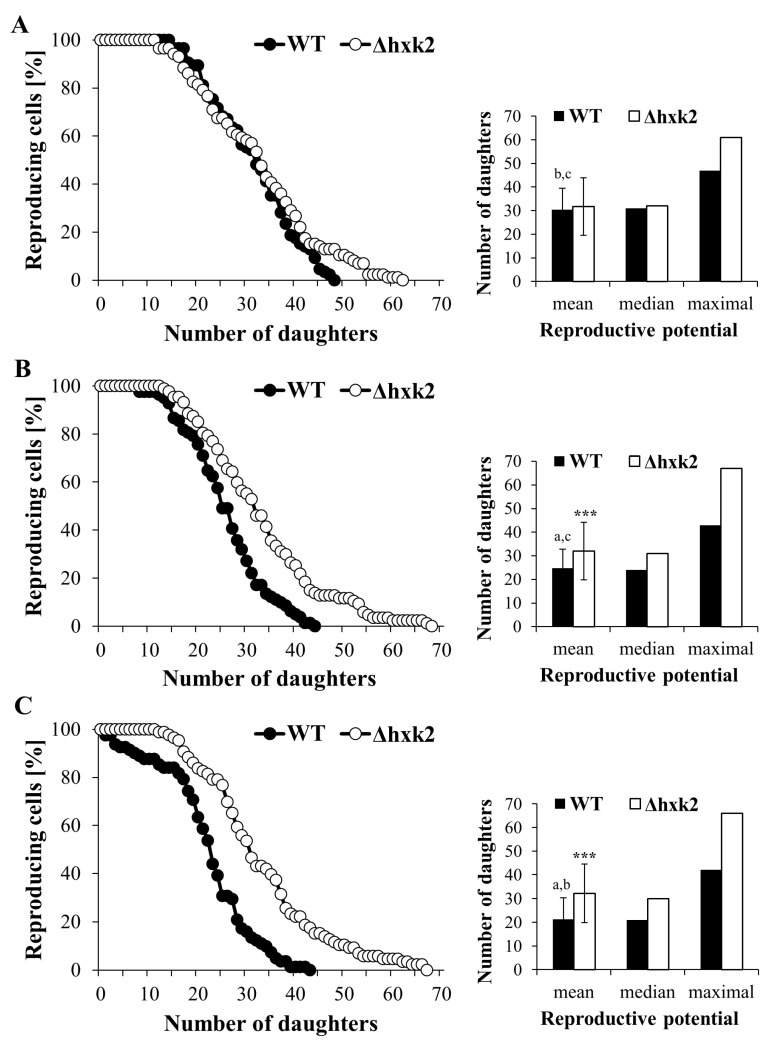
Reproductive potential of yeast cells cultured in conditions with different glucose concentration. (**A**) Reproductive potential of yeast cells growing in calorie restriction (CR) conditions, (**B**) reproductive potential of yeast cells growing in optimal conditions (**C**) reproductive potential of yeast cells growing in calorie excess (CE) conditions, The data represent the mean values from two independent experiments of 40 cells each. *** *p* < 0.001 as compared with the WT strain; a—different to medium with 0.5% glucose, b—different to medium with 2% glucose, c—different to medium with 4% glucose.

In order to explore the links between central carbon metabolism and proliferative capacity of the cells, analyses of reproductive potential in Δ*hxk2* mutant under conditions of different glucose concentration (CR—0.5%; optimal—2% and calorie excess (CE)—4%) were performed. Analysis of the reproductive potential showed that in general the Δ*hxk2* strain was able to produce more daughter cells in comparison to the WT strain (Figure 1A–C). Besides differences in the values of the maximum reproductive potential (47 for WT and 61 for Δ*hxk2* strain) under CR conditions, there were no observable differences in the reproductive potential between the Δ*hxk2* and the WT strains (Figure 1A). The mean value of that parameter in both strains was comparable, around 31 generations (30.5 for WT and 31.7 for Δ*hxk2* strain) (Figure 1A). In turn, differences in the reproductive potential between the WT and the Δ*hxk2* strains appeared with the increase in glucose concentration and were most noticeable in the case of CE conditions (Figure 1B–C). The mean value of the reproductive potential under CE conditions observed for the Δ*hxk2* strain was one and a half times higher than in case of the WT strain (Figure 1C). It should be emphasized that the reproductive potential of the Δ*hxk2* strain did not change between conditions with different glucose concentration (Figure 1A–C). Hence, the difference between the WT and Δ*hxk2* strains is the result of the decreased reproductive potential observed for the WT strain under conditions with higher glucose concentration, in particular under CE conditions (Figure 1C). These results are in agreement with our previous findings [7] and support the assumption that the Δ*hxk2* strain can be treated as a CR-mimetic strain.

### 2.2. Increased Respiration in the Case of Absence of Hexokinase 2 Is Partly Caused by Changes in the Metabolic Flux

**Figure 2:**
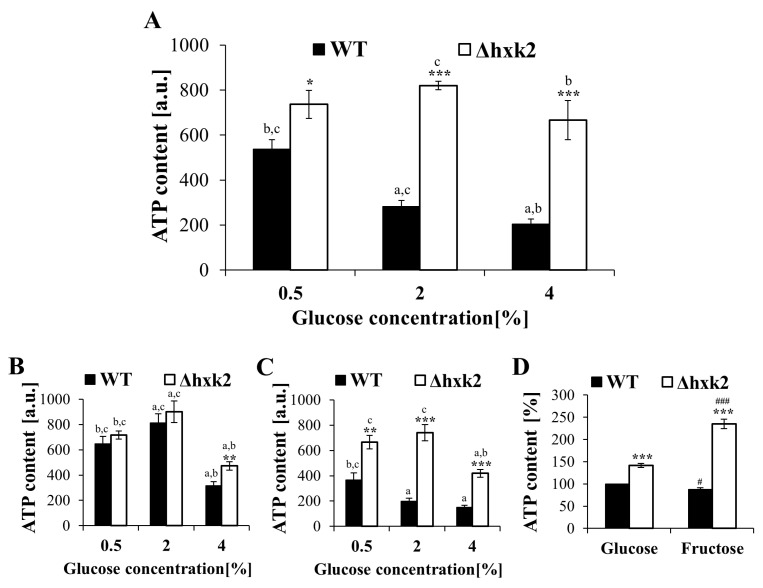
ATP content of yeast cells cultured in different metabolic conditions. (**A**) ATP content determined during exponential phase of growth, (**B**) ATP content determined after depletion of glucose in the medium and diauxic shift, (**C**) ATP content determined in respiratory-deficient (rho^0^) yeast cells during exponential phase of growth, (**D**) comparison of ATP in yeast cells cultured in medium with glucose or fructose used in the same 2% concentration. The results are presented as mean ± SD from three independent experiments. * *p* < 0.05, ** *p* < 0.01, *** *p* < 0.001 as compared to the WT strain; # *p* < 0.05, ## *p* < 0.001 as compared to the medium with glucose; a—different to medium with 0.5% glucose, b—different to medium with 2% glucose, c—different to medium with 4% glucose.

**Figure 3:**
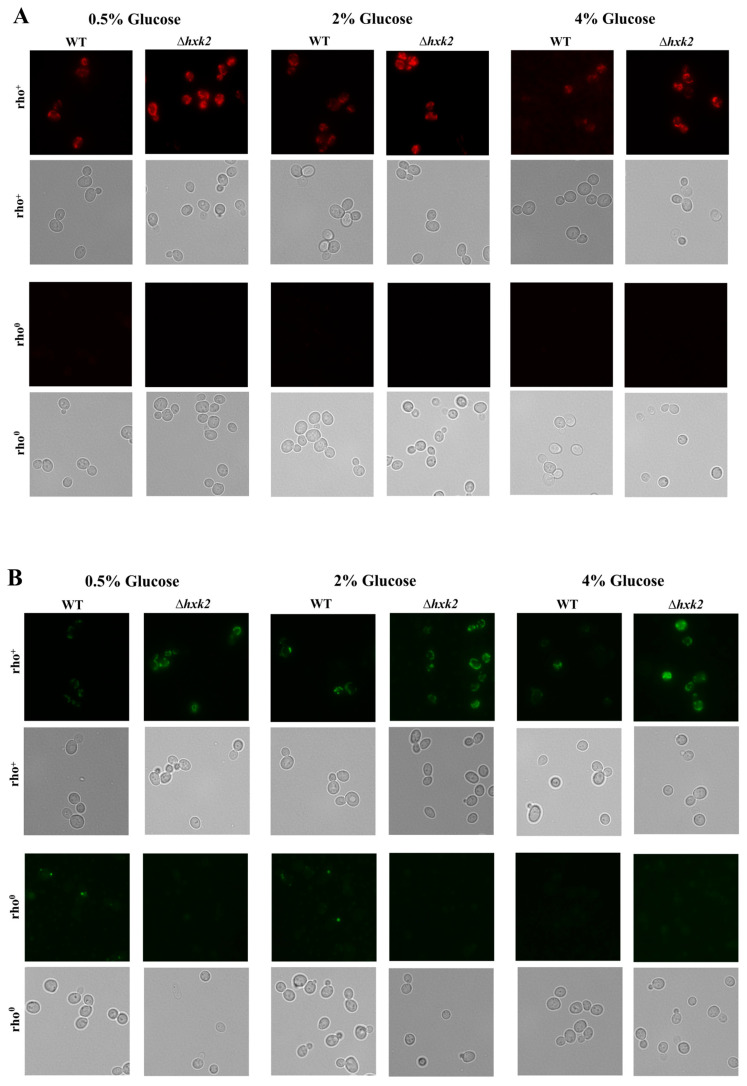

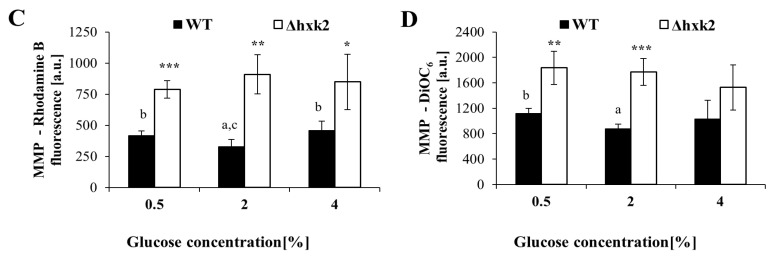
Mitochondrial membrane potential and morphology of the mitochondrial network of yeast cells from the early exponential phase of growth cultured in different metabolic conditions. (**A**) Morphology of the mitochondrial network and (**C**) mitochondrial membrane potential (MMP) determined using rhodamine B hexyl; (**B**) morphology of the mitochondrial network and (**D**) MMP determined using DiOC_6_(3). The fluorescence was measured at λ_ex_ = 555 nm and λ_em_ = 579 nm for rhodamine B or at λ_ex_ = 476 nm and λ_em_ = 501 nm for DiOC6(3). The results are presented as mean ± SD from three independent experiments. * *p* < 0.05, ** *p* < 0.01, *** *p* < 0.001 as compared to the WT strain; a—different to medium with 0.5% glucose, b—different to medium with 2% glucose, c—different to medium with 4% glucose. Mitochondrial network was visualized using fluorescence microscope Olympus BX-51 equipped with the DP-72 digital camera and cellSens Dimension v1.0 software at appropriate wavelengths. The microscopic images present typical results from of the duplicate experiment. Magnification 100×.

It is generally known that hexokinase 2 plays a crucial role in the regulation of glucose repression signal, which is the reason why the Δ*hxk2* mutant strain exhibits an increased rate of respiration [36,38]. To analyze whether lack of hexokinase 2 affects reproductive potential only by increasing respiration, or whether this is an overall action connected with fluxes in central carbon metabolic pathways, we investigated the changes in intracellular ATP content in cells of the Δ*hxk2* strain growing under different metabolic conditions (Figure 2A–D). Upon the exponential phase of growth in the WT strain, the highest level of ATP was noted in the case of cells growing on the medium with 0.5% glucose. In turn, in media with 2% and 4% glucose, in which yeast cells conduct fermentation, the ATP level was lower (Figure 2A). Therefore, in the case of the Δ*hxk2* strain, an increased level of ATP in comparison to the WT strain was observed in all glucose concentrations used. Most importantly, the level of ATP was similar in the cells of Δ*hxk2* strain cultivated in the conditions with low or high glucose concentrations (Figure 2A). Thus, deletion of *HXK2* indeed resulted in an increase in respiration. Nevertheless, it should be underlined that a high level of ATP in cells lacking hexokinase 2 is independent of the content of glucose in the medium. This is observed even under conditions where high glucose levels would indicate that metabolism should be fermentative. Hence, analyses, which allow us, to some extent, to determine the origin of ATP, were performed. Analysis of ATP content in the cell after diauxic shift (in the phase when cells due to glucose exhaustion use only aerobic respiration) showed that level of ATP in the Δ*hxk2* strain was similar in the growth conditions of 0.5% and 2% glucose concentrations, with the level noticed in the exponential phase (Figure 2A,B). A slight decrease in the ATP level in the Δ*hxk2* strain was observed in the conditions of CE between the exponential phase of growth and after diauxic shift (Figure 2A,B). On the other hand, in the case of the WT strain the ATP content after diauxic shift was significantly higher than the level observed under exponential phase of growth. Moreover, increased values of ATP content in the WT strain were comparable with those obtained for the Δ*hxk2* strain (Figure 2B). These results suggest that the ATP level, and thus respiration efficiency, may have an “upper limit” associated with cellular capacity. To get a better overview, the ATP levels were also checked in respiratory-deficient (rho^0^) yeast cells (Supplementary Materials Figure S1). Although, in general, the ATP level in respiratory-deficient cells was lower than the level noted for respiratory-competent cells, surprisingly the ATP content in rho^0^ cells lacking hexokinase 2 was significantly higher than the ATP content in rho^0^ WT cells (Figure 2C). For that reason, the mitochondrial membrane potential (MMP) and mitochondrial network morphology were also analyzed Figure 3 shows a rhodamine B and DiOC_6_ spectrofluorometric measurement and microscopic labeling experiments. Both of these dye are taken up by mitochondria in an MMP-dependent manner. The MMP of Δ*hxk2* cells was similar in all tested conditions (different glucose concentration in medium) and, in all cases, was higher compared with WT strain (Figure 3C,D). Similarly, mitochondrial network was more developed in the Δ*hxk2* cells in comparison to the WT strain (Figure 3A,B). The rho^0^ cells did not show any fluorescent signal, presumably due to loss of membrane potential, in which case the dye cannot accumulate (Figure 3A,B). This suggests that the higher level of ATP in the Δ*hxk2* strain is not only the result of respiration efficiency. Considering the role of Hxk2p in cellular metabolism, the possible explanation for this phenomenon may be connected with fluxes in central carbon metabolic pathways, as indicated by the cellular level of ATP in the medium with glucose or fructose. The level of ATP was similar in the case of the WT strain but significantly different in the case of the Δ*hxk2* strain; it was more than one and a half times higher in the medium with fructose than in the medium with glucose (Figure 2D). It is worth noting here that both glucose and fructose are treated as fully fermentable carbon sources.

### 2.3. Absence of Hexokinase 2 Slightly Decreases the Growth Rate and Vitality of the Cell

**Figure 4:**
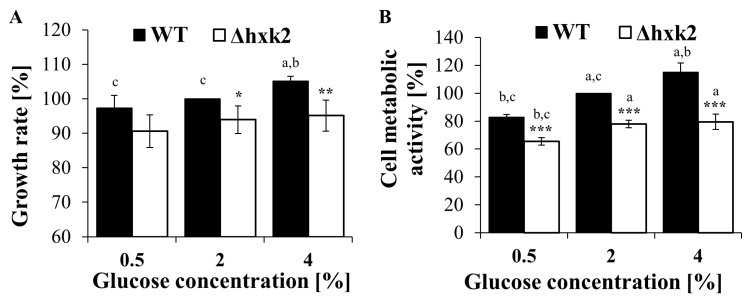
The growth of the yeast cells population and metabolic activity of yeast cells cultured in conditions with different glucose concentration. (**A**) The growth rate determined on the basis of growth kinetic of yeast cells population; (**B**) overall metabolic activity (an equivalent of cell vitality) of the cell determined with FUN-1 stain. The results are presented as mean ± SD from three independent experiments. * *p* < 0.05, ** *p* < 0.01, *** *p* < 0.001 as compared to the WT strain; a—different to medium with 0.5% glucose, b—different to medium with 2% glucose, c—different to medium with 4% glucose.

As glucose is used in several cross-linking intracellular pathways, each disorder in glucose metabolism can affect physiological efficiency and vitality of the cell. One of the determinants of physiological fitness of the yeast cell is the rate of cell population growth. There were noticeable differences in growth rate both between different glucose concentration conditions and between strains. Cells growing in CR conditions showed the lowest growth rate. In the case of the WT strain, the growth rate slightly increased with increasing glucose concentration and was the highest for cells growing under CE conditions. On the other hand, the growth rate in the case of the Δ*hxk2* strain was significantly lower in comparison to the WT strain in conditions with 2% and 4% glucose concentrations (Figure 4A). The analysis of FUN-1 staining, which enables estimation of the overall vitality and metabolic activity of cells, showed similar relationships, both between the analyzed strains and between different glucose concentration conditions. In the case of the Δ*hxk2* strain, the overall cell vitality status was lower than that observed in the WT strain, and the values of that parameter were similar between conditions with different glucose concentrations. Inversely, in the case of the WT strain, increased values of vitality status were observed with increasing glucose concentration (Figure 4B). These observations demonstrate that deletion of *HXK2* slightly decreases the overall physiological efficiency of yeast cells, even though they possess a higher-than-usual level of ATP. However, it should be emphasized that physiological efficiency of the cell depends not only on the energy capabilities of the cell but also on its biosynthetic capacity.

### 2.4. Cells Devoid of Hexokinase 2 Feature Smaller Size and Reduced Biosynthetic Capabilities

**Figure 5:**
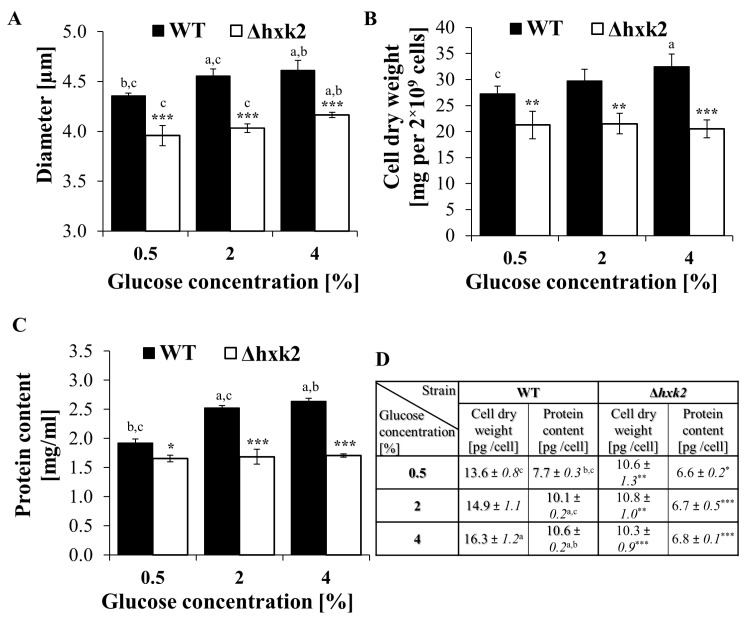
Parameters connected with biosynthetic capabilities of yeast cells cultured in conditions with different glucose concentration. (**A**) The cell size of yeast cells cultured in conditions of different glucose concentrations. Diameter of the cells was estimated through analysis of microscopic images using the cellSens Dimension software; *n* = 300 cells; (**B**) cell dry weight determined using moisture analyzer. The data present cell dry mass from three independent experiments where 2 × 10^9^ cells each were used; (**C**) protein content in cell extracts obtained from strictly defined number of cells (5 × 10^8^ yeast cells from the exponential phase culture); (**D**) comparison of cell dry weight and protein content expressed in the same unit per cell. The results are presented as mean ± SD from three independent experiments. * *p* < 0.05, ** *p* < 0.01, *** *p* < 0.001 as compared to the WT strain; a—different to medium with 0.5% glucose, b—different to medium with 2% glucose, c—different to medium with 4% glucose.

Cell size and cellular biosynthetic efficiency are important factors influencing yeast reproductive potential [9,39]. Therefore, to verify the assumption that the results noted in the Δ*hxk2* strain are caused by changes in its biosynthetic capabilities, cell size, cell dry weight, and protein content per cell were determined. It was noted that cells of the Δ*hxk2* strain have significantly smaller mean cell size in comparison to WT cells in all of the analyzed conditions (Figure 5A). Moreover, the differences in mean cell size in the Δ*hxk2* strain between conditions with different glucose concentrations were minor. Only in the CE conditions was a larger mean cell size of the Δ*hxk2* strain observed. Even in those conditions, however, the mean cell size of the Δ*hxk2* strain was significantly smaller in comparison to cells of the WT strain, including WT cells growing in CR conditions (Figure 5A). In the case of the WT strain, mean value of cell size increased with increasing glucose concentration, and the largest mean cell size was noted in the medium with 4% glucose concentration (Figure 5A). As the cell size is directly connected with the level of biosynthesis, significant differences in cell dry weight and protein content between yeast strains were observed. In the case of the WT strain, the lowest value of cell dry weight was observed under CR conditions and gradually increased with the increase in glucose concentration (Figure 5B,D). In turn, such an increase in the value of that parameter was not observed for the Δ*hxk2* strain. Cell dry weight in the Δ*hxk2* strain was significantly lower in comparison to the WT strain and was not changed between different glucose concentration conditions (Figure 5B,D). Proteins usually make up just over half of the cellular content; therefore, changes in protein content clearly correlate with the changes in cell dry weight (Figure 5B,D) and cell size results (Figure 5A). Increase in cell dry weight is accompanied by increase in protein content. Cells growing in the CR conditions showed the lowest protein content. In the case of the WT strain, the level of protein content gradually increased with increase in the glucose concentration and was the highest for cells growing in CE conditions. At the same time, protein content in the Δ*hxk2* strain was unchanged for different glucose concentration conditions and was significantly lower in comparison to the WT strain (Figure 5C,D). Results of cell dry weight and protein content evaluated as a quantity calculated per cell (Figure 5D) showed that the level of available glucose has an influence on cell biosynthesis efficiency mainly through changes in the amount of protein in the cell. The obtained results displaying specific biosynthetic efficiency of cells under conditions with different glucose concentrations (Figure 5A–D) and the opposite results for reproductive potential of the yeast cell (Figure 1A–C) confirm that there is a close relationship between cell biosynthetic efficiency, size, and reproductive capacity.

### 2.5. Metabolic Trade-Off in the Case of Absence of Hexokinase 2 Is Connected with Alteration in the Pentose Phosphate Pathway

**Figure 6:**
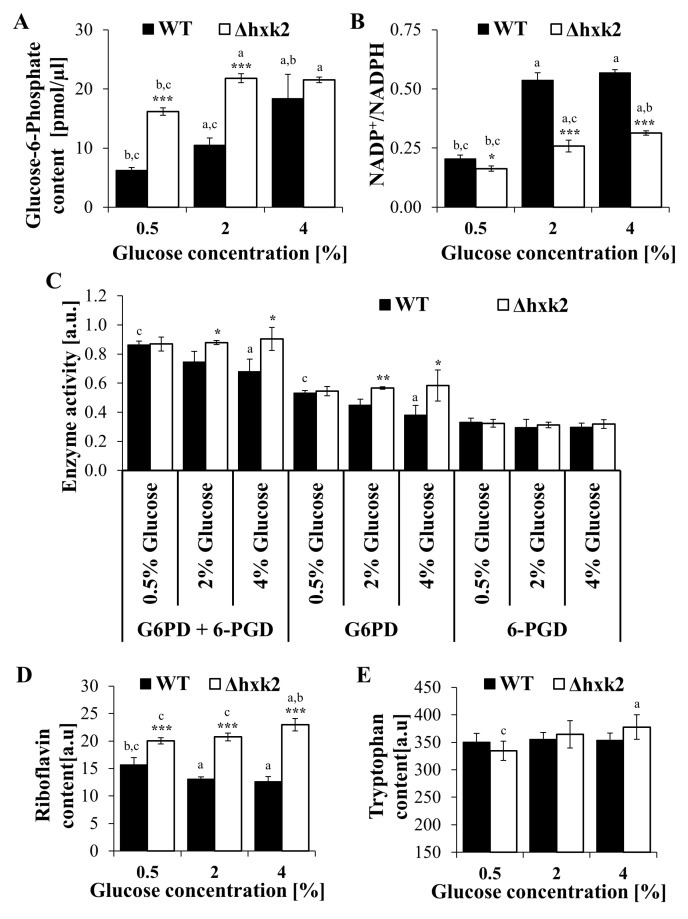
Parameters connected with biosynthetic capabilities and the action of the pentose phosphate pathway in yeast cells cultured in conditions with different glucose concentration. (**A**) The content of glucose-6-phosphate (G6P) determined in deproteinized cell extracts (10 kDa MWCO) using Glucose-6-Phosphate Assay; (**B**) NADP^+^, and NADPH contents determined with the use of NADP/NADPH-Glo Assay. The results are presented as a NADP^+^/NADPH ratios; (**C**) pentose phosphate (PP) pathway enzymes activity: glucose-6-phosphate dehydrogenase (G6PD) and 6-phosphogluconate dehydrogenase (6-PGD) determined spectrophotometrically by measuring the rate of NADP+ reduction at 340 nm. The data was expressed in arbitrary units as mean ± SD, (**D**) riboflavin content in the cell extracts determined by fluorimetric measurements at λ_ex_ = 460 nm and λ_em_ = 535 nm. The values are expressed in arbitrary units, (**E**) tryptophan content in the cell extracts determined by fluorimetric measurements at λ_ex_ = 290 nm and λ_em_ = 325 nm. The values are expressed in arbitrary units. The results are presented as mean ± SD from three independent experiments. * *p* < 0.05, ** *p* < 0.01, *** *p* < 0.001 as compared to the WT strain; a—different to medium with 0.5% glucose, b—different to medium with 2% glucose, c—different to medium with 4% glucose.

Glucose apart from being of energetic importance provides the carbon skeleton for macromolecule biosynthesis. An important part in this process is played by the pentose phosphate pathway (PP pathway), which through the use of glucose-6-phosphate (G6P) as a substrate and common intermediates is closely linked to glycolysis. This causes that glucose can be used by several cross-linking intracellular pathways, which enables cells to adapt metabolism to the current energetic and biosynthetic needs. Considering the above and also the differences in the level of ATP (Figure 2A–D) and biosynthetic capabilities of the cells (Figure 5A–D), the study examined the way in which absence of hexokinase 2 influenced on parameters connected with the PP pathway. The tested parameters included the content of G6P, changes in the NADP(H) pool, PP pathway enzymes activity, and the content of riboflavin and tryptophan. The content of G6P was significantly higher in the Δ*hxk2* strain in comparison to the WT strain. Moreover, G6P content in Δ*hxk2* cells was high in all tested conditions (Figure 6A). In turn, in the case of WT strain cells, the level of G6P was the lowest in CR conditions and gradually increased with the increase in glucose concentration. Although the G6P content results in the WT strain were anticipated, the high level of G6P in Δ*hxk2* strain was less obvious. Due to the fact that the Δ*hxk2* strain has a higher level of PP pathway substrates, we examined the level of NADP(H) cofactors as products of the PP pathway and changes in the NADP(H) pool. There were only slightly differences in the level of NADPH between cells of the WT and Δ*hxk2* strains, but, at the same time, distinct differences were noted in the level of NADP^+^ (the level of NADP^+^ was significantly lower in the Δ*hxk2* strain) (data not shown). Analysis of NADPH and NADP^+^ levels showed that absence of Hxk2p and different glucose concentrations substantially change the share of NADPH and NADP^+^ in the total NADP(H) pool (Figure 6B). It was noted that the NADP^+^/NADPH ratio was the lowest in both strains in the CR conditions. Under 2% and 4% glucose concentration conditions, an increased level of NADPH oxidation resulting in a higher NADP^+^/NADPH ratio was observed, although that ratio was still significantly lower in cells of the Δ*hxk2* strain in comparison to the WT strain (Figure 6B). This suggests a lower usage of NADPH for cellular demands in cells of the Δ*hxk2* strain, including biosynthesis requiring NADPH as a reducing agent. Furthermore, this suggestion seems to be confirmed by the results of the analysis of yeast cells’ biosynthetic capabilities (Figure 5A–D). Given that both the content of G6P and changes in NADP(H) pool directly influence the overall PP pathway activity, analysis of the PP pathway enzyme activity (G6PD and 6-PGD) was performed. It was observed that the activity of the PP pathway enzymes in cells growing under CR conditions was similar in both strains. Cells showed similar activity in both G6PD and 6-PGD enzymes (Figure 6C). With increasing glucose concentration differences in activity of the analyzed enzymes became visible between strains. There were no differences in the analyzed enzymes activity under conditions with 0.5%, 2%, and 4% glucose concentrations in the case of the Δ*hxk2* strain. In turn, in the case of the WT strain, although the activity of 6-PGD was not changed, a distinct reduction of the G6PD and total dehydrogenase activity was observed with the increased glucose concentration. However, it is worth underlining that both strains had similar 6-PGD activity, while differing in the G6PD activity (Figure 6C). Given that the PP pathway activity is considered an important cellular biosynthetic route of glucose utilization and taking into the account the cell biosynthetic capability results (Figure 5A–D), the observed activity of PP pathway enzymes in the case of the Δ*hxk2* strain was unpredictable. It should be emphasized that the PP pathway is not only a source of aromatic amino acids precursors and NADPH but also a provider of ribose-5-phosphate used in nucleotides biosynthesis, including nucleotides not involved in DNA replication, such as IMP, ATP, or GTP. Hence, higher activity of PP pathway enzymes in the case of the Δ*hxk2* strain (Figure 6C) seems to provide ribose-5-phosphate for exceptionally high ATP synthesis observed in this strain (Figure 2A–D). To verify that possibility, determination of riboflavin and tryptophan content was performed as the biosynthesis of riboflavin and tryptophan based on ribose-5-phosphate and other PP pathway intermediates [40]. There was a significantly higher level of riboflavin in cells of the Δ*hxk2* strain in comparison to the WT strain under all conditions utilizing different glucose concentrations (Figure 6D). Moreover, in the case of the Δ*hxk2* strain, the level of riboflavin slightly increased with increase in glucose concentration. In turn, in the case of the WT strain, the opposite tendency was noted, i.e., the level of riboflavin decreased with increase in glucose concentration (Figure 6D). In the case of tryptophan content, in general, there were no differences either between strains or individual glucose concentration conditions. Statistically significant differences were noted only between cells of Δ*hxk2* strain growing in media with 0.5% and 4% glucose (Figure 6E). These observations confirm that higher activity of PP pathway enzymes in the case of the Δ*hxk2* strain is connected with higher demands for ribose-5-phosphate in this strain.

### 2.6. Regardless of the Availability of Glucose, Absence of Hexokinase 2 Significantly Increases Proteasomal Activity of the Cell

**Figure 7:**
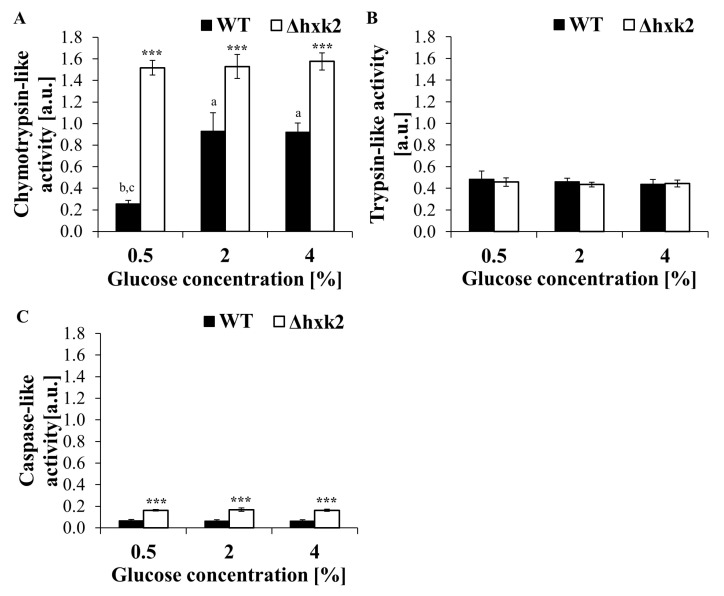
Proteasomal activity in yeast cells cultured in conditions with different glucose concentration. (**A**) Chymotrypsin-like activity determined with Suc-LLVY-AMC fluorogenic peptide, (**B**) trypsin-like activity determined with Z-ARR-AMC fluorogenic peptide; (**C**) caspase-like activity determined with Z-LLE-AMC. The degradation of fluorogenic peptide was measured by monitoring the fluorescence of the reaction product, free 7-amino-4-methylcoumarin (AMC). The rate of fluorescence increase was measured at λ_ex_ = 350 nm and λ_em_ = 440 nm. The values are expressed in arbitrary units. *** *p* < 0.001 as compared to the WT strain; a—different to medium with 0.5% glucose, b—different to medium with 2% glucose, c—different to medium with 4% glucose.

Proliferative capacity of cells seems to be associated with proper protein turnover. Decrease in the activity of protein quality control mechanisms is believed to be one of the proposed causes of the age-related cellular dysfunction. One of the protein control mechanisms is its degradation, which can be carried out by autophagy or by the ubiquitin proteasome system. The proteasome exhibits three different types of activity: chymotrypsin-like, trypsin-like, and caspase-like. The level of these activities depends on the protein substrate and allosteric interactions between them, which allows for strict regulation of proteolytic functions in response to changes in the cellular metabolism. Therefore, to verify the possibility that changes in biosynthetic capabilities and metabolic alteration observed in the Δ*hxk2* strain are accompanied by changes in proteasomal activity, three distinct proteasomal peptidase activities (chymotrypsin-like, trypsin-like, and caspase-like) were tested. It was observed that cells of the Δ*hxk2* strain have significantly higher chymotrypsin-like and caspase-like proteasomal activities in comparison to WT cells under all of the analyzed conditions (Figure 7A,C). There were no differences in trypsin-like activity between Δ*hxk2* and WT strains (Figure 7B). There were also no differences in each proteasomal peptidase activities between conditions with different glucose concentrations, apart from the difference in chymotrypsin-like activity between conditions with 0.5% and 2% or 4% glucose observable in the case of the WT strain (Figure 7A–C). It should be also emphasized that, in yeast cells, the chymotrypsin-like activity has the largest impact on the overall proteasome activity (Figure 7A–C). These results demonstrate that cells lacking Hxk2p display increased proteasome activity regardless of the availability of glucose, which, together with lower biosynthetic capabilities may suggest improvement in proteostasis.

## 3. Discussion

Cellular metabolism is tightly controlled and responds to changes in extracellular conditions and intracellular demands. Carbohydrate utilization is the central metabolic pathway providing energy and building blocks to the cell. The importance of this process is indicated by (1) association between the number of chronic diseases and abnormal regulation of carbohydrate metabolism, e.g., diabetes; (2) negative impact of high sugar conditions on the organism’s physiology and acceleration of aging in certain species; and (3) improvement of the health span and functionality in a wide range of organisms by calorie restriction (CR) [14,41].

One of the most often postulated mechanisms of CR action in the case of studies using yeast cells is that CR extends yeast lifespan by increasing respiration [42,43,44]. However, findings showing that CR increases lifespan also in respiratory-deficient yeast cells (rho^0^) challenge the validity of that assumption [25,26]. In the case of yeast, CR is obtained by reducing the glucose concentration in the medium; therefore, the role of hexose kinases in the glucose metabolism explains the use of the strain lacking *HXK2* as a genetic model of CR. Consistently with previous findings [43,45], cells lacking *HXK2* display increased reproductive potential (Figure 1B,C) together with increased respiration (evidenced by the high level of ATP, high value of MMP and developed mitochondrial network—Figure 2A,B and Figure 3A–D). However, other observations in the case of Δ*hxk2* strain, such as decreased rate of glucose uptake [12]; disturbing in glucose repression even in high glucose media (this study); the MMP analysis; mitochondrial morphology; and ATP level, in used conditions and rho^0^ cells (Figure 3A–D and Figure 2A–D) led to the assumption that increased reproductive potential (Figure 1A–C) is not a direct result of increased respiratory metabolism. But it may be a consequence of changes in metabolic fluxes in central carbon metabolic pathways. Presumably, deletion of *HXK2* substantially changes the metabolic flux in carbon metabolism pathways, in which only one of the effects is increased respiration. Such an assumption is in line with the results showing that glucose repression requires sufficient glucose transportation and high glycolytic flux [46], as well as would also explain the issue of the extended lifespan in rho^0^ cells. Therefore, high level of ATP in the Δ*hxk2* strain, apart from increased respiration, may also result from (i) lower usage of ATP; (ii) differences in metabolic trade-off between glucose utilization pathways; and (iii) ability to regulate metabolic pathways through the produced metabolites, especially the glycolytic metabolites.

These assumptions are closely connected with the activity of cAMP/PKA pathway, in which activation in glucose-dependent manner stimulates glycolytic flux, represses expression of respiratory metabolism genes, induces ribosome biogenesis and suppresses stress response [29,47]. Therefore, there is evidence that the Hxk2p-dependent glucose repression pathway overlaps with the cAMP/PKA signaling pathway; among others are intracellular sugar phosphorylation and the related high glycolytic flux are necessary for activation of the cAMP/PKA pathway [45,48]. Hence, lack of *HXK2* resulting in lower activity of the cAMP/PKA pathway may influence the level of ATP on the one hand due to derepression of respiratory metabolism genes but from the other due to lower usage of ATP for ribosome biogenesis, protein biosynthesis and synthesis of cAMP. What is more, yeast cell cycle progression requires effective glycolysis and the impaired fermentation, may decreased the expression of *CLN3* and *CDC28* genes [49]. The impaired fermentation in the case of the Δ*hxk2* strain has been confirmed by our results (Figure 3A–D). Furthermore, the reproductive potential data observed in the Δ*hxk2* strain (Figure 1A–C) and in the previously analyzed Δ*gpa2* and Δ*grp1* strains point out to a significant role of the cAMP/PKA pathway in proliferative capacity of the cell. It is worth mentioning that, besides genetic alteration, lower activity of the cAMP/PKA pathway can also be obtained by lower availability of glucose concentration [7] and (this study).

A higher ATP level can also be partly caused by changes in CCM pathways, especially given that some literature data seem to indirectly confirm such idea. First, it was shown that deletion of *HXK2* results in decreased fluxes through the glycolytic/fermentative enzymes and significant reduction in fermentative capacity [12,38,50]. Secondly, flux through the tricarboxylic acid (TCA) cycle can be independent of glucose concentration of the medium, but is inversely correlated with glucose uptake rate and to a lesser extent with the growth rate [51]. Indeed, in the case of the Δ*hxk2* strain, significantly lower glucose uptake [12] and lower growth rate (this study) were observed. Next, the metabolic reaction observed in the Δ*hxk2* strain is closely related to the Crabtree effect and the phenomenon of trade-off between overlapping pathways of carbon metabolism. Our studies suggest that the connection between energy-producing and biomass formation pathways is generally closed in the metabolic triangle: the respiration—glycolysis—pentose phosphate pathway. An important role in this relationship is played by glycolytic flux associated with the glucose uptake rate. On the one hand, high glycolytic flux will favor fermentation and production of building blocks; on the other hand, it will reduce respiration capabilities. Hence, it is observed that ATP is produced by respiration at low glucose uptake rate [52,53], which is consistent with our observations (this study) and [12]. It turn, high glucose uptake rate and high glycolytic flux will correspond to higher biosynthetic rate. In fact, such a correlation has been observed in all parameters showing biosynthetic capabilities of cells (i.e., cell size, cell dry weight, protein content) (Figure 5A–D). Cells of the Δ*hxk2* strain which exhibit lower glucose uptake rate, lower fermentation yield [12], decreased growth rate, increased respiration metabolism, and higher level of ATP (this study; Figure 2A–D, Figure 3A–D and Figure 4A,B) in comparison to the WT strain show essentially low biosynthetic capabilities (Figure 5A–D). Remarkably, low biosynthetic capabilities may additionally explain a high level of ATP in the Δ*hxk2* strain due to the fact that about half of the energy generated in CCM is used for macromolecular synthesis, especially for protein synthesis [34]. Moreover, the assumption of changes in the CCM pathway seems to be confirmed by the results of ATP content in cells grown on the medium with fructose. In yeast cells grown on non-fermentable carbon sources or lacking *HXK2*, a de-repression and production of Hxk1p and Glk1p is observed [37]. Therefore, similar results of the ATP level in the WT strain and a significant difference in ATP levels between glucose and fructose conditions in the case of the Δ*hxk2* strain (Figure 2D) cannot be explained by an increase in respiration due to removal of the respiratory repression effect because of the fact that Hxk1p can maintain respiration metabolism repression [54]. Due to the fact that Hxk1p is a negative regulator of *GLK1* expression and while Glk1p is not able to phosphorylate fructose, it is suggested that the ATP level in cells growing in a medium with fructose is a result of high activity of Hxk1p. This is consistent with the previous findings presenting higher fructose-phosphorylating capacity in the Δ*hxk2* strain [38].

An important part in controlling the metabolic flux is played by intracellular metabolites, especially glycolytic intermediates. The role of fructose 1,6-bisphosphate (F1,6bP) as a regulator of respiratory activity is well known. F1,6bP directly inhibits the activity of complex II and III of the respiratory chain, but its level also correlates with the glucose uptake rate, and high F1,6bP content indicates high glycolytic flux [34,53,55]. It is worth underlining that a lower concentration of F1,6bP is observed in the Δ*hxk2* strain cells [38,55], although as shown by the latest research, induction of the Crabtree effect and regulation of respiration also depend on the concentration of glucose-6-phosphate (G6P), which is regarded as a stimulator of the respiratory chain [55]. The level of G6P in the case of the Δ*hxk2* strain was high in all conditions with different glucose concentrations (Figure 6A). The observed high level of G6P (this study) together with the low level of F1,6bP [38,55] lead to high G6P/F1,6bP ratio what result in increased respiratory activity. This is confirmed by both the results of ATP content (Figure 2A–D) and the literature data showing that the correspondingly low G6P/F1,6bP ratio (below 0.8) is responsible for respiratory chain inhibition and induction of the Crabtree effect [55]. Nevertheless, the reason for such a high concentration of G6P in cells lacking the main hexokinase is puzzling. This may result from (i) increased activity of Glk1p (in the absence of Hxk2p), which is not feedback inhibited by G6P; and (ii) accumulation of glycolytic intermediates (including pyruvate) as a result of saturated mitochondrial ATP production capacity [53]. The latter possibility seems to be confirmed by the results showing an intracellular accumulation of pyruvate in the Δ*hxk2* strain [38].

It is important to note that G6P is also the starting point of the PP pathway, which is an important pathway of cellular biosynthesis. Anabolic reactions require oxidation of NADPH to NADP+ and the main route for NADPH production is the PP pathway. Although no significant differences in NADPH production were observed, a significantly lower usage of NADPH was noted in the case of the Δ*hxk2* strain, which was evidenced by the low ratio of NADP+ to NADPH (Figure 6B). This suggest the low usage of NADPH for the biosynthesis of components forming the cell’s biomass, which is consistent with results of overall biosynthetic capabilities in the Δ*hxk2* strain (Figure 5A–D), but also with the previous results obtained in yeast [7] and cell lines [56]. Therefore, it was expected that the activity of PP pathway enzymes in the Δ*hxk2* strain should be low; despite expectations, a remarkably high activity of the PP pathway enzymes, independent of glucose concentration, was observed in the Δ*hxk2* strain (Figure 6C). Nevertheless, it must be emphasized that the PP pathway and the related glycolysis are regulated depending on cellular demands. Moreover, the PP pathway is not only responsible for production of macromolecular components, such as aromatic amino acids, NADPH, or nucleotides involved in DNA replication, but it also provides ribose-5-phosphate for the de novo synthesis of glucose-derived intermediates, including IMP, ATP, GTP, riboflavin, or tryptophan [40]. Thus, higher activity of PP pathway enzymes in the case of Δ*hxk2* strain (Figure 6C) would provide ribose-5-phosphate for exceptionally high ATP level observed in this strain. Such possibility is confirmed by the results of high content of other ribose-5-phosphate-dependent metabolites (Figure 6D,E) and the literature data presenting slightly higher concentration of AMP and ADP in the case of Δ*hxk2* strain [38]. Changes in the parameters directly connected with glucose metabolism and high level of ATP observed in ∆*hxk2* mutant cells, both with or without functional mitochondria, indicate that cells lacking Hxk2p are caught in a metabolic loop that makes them resistant to carbon source availability. Hence, in view of the fact that the growth rate of a yeast cells is controlled by TORC1-PKA signaling circuit [57], the high level of ATP coupled with lower activity of cAMP/PKA pathway may block the activation of TORC1 controlled transcriptional programs.

The metabolic changes are also important in the context of cellular biosynthetic possibilities what has a crucial impact on the reproductive potential of the cell. This work and our previous findings [7] demonstrate that alterations decreasing the overall biosynthetic capabilities, protein production and size of the cell increase reproductive potential of the cell. Moreover, the results obtained with the use of the Δ*hxk2* strain confirm the positive impact of low glycolytic flux and decreased PKA-activity on cell reproductive potential [7,58,59], and additionally complement the above explanations. The correlation noted between cell size and reproductive potential in the case of the Δ*hxk2* strain is consistent with the hypertrophy hypothesis which assumes that cell size, and in particular the rate of cell size increase per generation, is an important regulator of the yeast cell’s reproductive potential [8,39]. Besides yeast, such a relationship is also observed for certain types of mammalian and human cells [60,61,62]. Hence, due to the fact that cell size is directly connected with biosynthetic processes, lower biosynthesis capabilities and the related decrease in cell size may lead to slower achievement of the hypertrophy state and result in extension of the proliferation capacity. The connection between biosynthetic capabilities, and decline in reproductive potential was noted in several studies. It has been observed that, among others, (i) replicatively aged yeast cells displayed overabundance of protein and loss of stoichiometry in protein complexes [63]; (ii) excess levels of unneeded proteins disturbed proliferation of the budding yeast [64]; (iii) depletion of ribosomal 60s subunits and decrease in ribosome biogenesis extended reproductive potential [65]; (iv) chaperone overexpression extended lifespan, including through the decrease of protein synthesis and the amount of misfolded proteins [66]; (v) overexpression of mRNA binding protein Ssd1p reduced translation and extended lifespan [67]; and (vi) lower biosynthetic capabilities caused by reduced cAMP-PKA activity obtained through loss of GPCR system proteins (Gpa2p and Gpr1p) or CR increased reproductive potential [7].

In the light of the above arguments and the results of our work, it is clear that increase in reproductive potential is connected with decrease of cell biosynthetic burden. Taking into account the fact that among all cellular macromolecules protein production is the largest biosynthetic burden, any alteration that results in reduced protein and overall biosynthesis seems to be beneficial for reproductive potential due to proteostasis improvement. Indeed, a growing body of studies indicates that there is a strict linkage between cellular proteostasis and reproductive capabilities and longevity. The main task of proteostasis is to maintain a functional proteome in the living cell [68]. An integral part of cellular proteostasis is the ubiquitin proteasome system, whereas its insufficient activity results with accumulation of damaged protein but also can lead to induction of programmed cell death. Some literature data suggest the existence of direct interaction between hexokinase and mitochondria. This interaction can be involved in the apoptosis process by modulation of cytochrome c release [69]. Nonetheless, there are results suggesting that such interaction can be important mainly in pro-apoptotic conditions, e.g., after treatment of the acetic acid [70].

Nevertheless deletion of *HXK2* increased proteasomal activity (Figure 7A–C), which, together, with other metabolic consequences, can be the reason for the increased reproductive potential noted in the case of the Δ*hxk2* strain (Figure 1A–C). The connection between elevated proteasome-mediated protein degradation and replicative lifespan extension in yeast cells is already known [71,72], although our research showed that increased proteasomal activity in the case of the Δ*hxk2* strain is maintained even under CE conditions. The studies of Yao et al. [72] demonstrating that increased proteasome activity may induce respiratory metabolism through the degradation of Mig1p support explanation of results concerning the high level of ATP observed in the Δ*hxk2* strain (Figure 2A–D). Furthermore, elevated proteasome-mediated protein degradation together with low biosynthetic capabilities provides better turnover and protein recycling, which is beneficial in conditions of nutrient deficiency. It also provides protection against the accumulation of non-functional and damaged proteins.

## 4. Materials and Methods

### 4.1. Chemicals

BacTiter-Glo^™^ Microbial Cell Viability and NADP/NADPH-Glo^™^ Assay Kits were from Promega (Madison, WI, USA). Z-Ala-Arg-Arg-7-amino-4-methylocoumarin (Z-ARR-AMC) was from Merck Millipore (Darmstadt, Germany). Suc-Leu-Leu-Val-Tyr-7-amino-4-methylocoumarin (Suc-LLVY-AMC) and Z-Leu-Leu-Glu-7-amino-4-methylocoumarin (Z-LLE-AMC) were from Enzo Life Sciences (Farmingdale, NY, USA). FUN^®^ 1 Cell Stain, rhodamine B hexyl ester and 3,3′-Dihexyloxacarbocyanine Iodide (DiOC6(3)) were from Molecular Probes (Eugene, OR, USA). Coomassie Protein Assay Reagent (Thermo Scientific, Rockford, IL, USA) All other reagents were purchased from Sigma-Aldrich (Poznan, Poland). Components of culture media were from BD Difco (Becton Dickinson and Company, Spark, MD, USA) except for glucose (POCH, Gliwice, Poland).

### 4.2. Yeast Strains and Growth Conditions

The following yeast strains were used: wild-type (WT) BY4741 *MAT*a *his3 leu2 met15 ura3* and Δ*hxk2* mutant isogenic to BY4741 *MAT*a *his3 leu2 met15 ura3*YGL253W::*kanMX4* (Euroscarf, Scientific Research and Development GmbH, Oberursel, Germany). Yeast was grown in the liquid YP medium (1% Yeast Extract, 1% Yeast Bacto-Peptone) with different glucose concentrations (0.5, 2 and 4%) on a rotary shaker at 150 rpm, at 28 °C.

### 4.3. Determination of Cell Reproductive Potential

Reproductive potential of yeast cells was determined by a routine procedure [73] on cells placed on agar plates using a micromanipulator, with modifications described in Reference [74]. One-microliter aliquots of overnight yeast cultures grown on YPD liquid medium with specified glucose concentration were dropped on YPD plates with solid medium containing specified concentration of glucose; 0.5%, 2%, and 4%, respectively. For each experiment, forty single cells were micromanipulated to the appointed area. The first daughters were chosen as the starting cells, and their successive buddings were followed to determine the reproductive potential. After last budding, yeast cells were inspected to determine the moment of cell death. During the manipulation, the plates were kept at 28 °C for 16 h and at 4 °C during the night. The data represent mean values from two separate experiments.

### 4.4. Assessment of the Cellular ATP Content

The level of ATP in yeast cells was determined with BacTiter-Glo^™^ Microbial Cell Viability Assay according to the manufacturer’s protocol (Promega) with own modifications. Cells from specified phase of growth (the early exponential and after diauxic shift) or specified conditions (grown on medium with glucose or fructose) were suspended in a 100 mM phosphate buffer with pH 7.0, containing 0.1% glucose and 1 mM EDTA. A sample of cell suspension with a density of 10^6^ cells/mL was used for determination purposes. The luminescent signal, proportional to the amount of ATP, was recorded using the TECAN Infinite 200 microplate reader (Tecan Group Ltd., Männedorf, Switzerland) after appropriate time (until the luminescence signal obtain stable level).

### 4.5. Determination of Mitochondrial Membrane Potential and Mitochondrial Network Morphology

Mitochondrial membrane potential (MMP) and morphology of the mitochondrial network were determined using rhodamine B hexyl ester and DiOC_6_(3). Cells from the early exponential phase of growth were washed twice with sterile water and suspended in a 20 mM HEPES buffer with pH 7.4, containing 5% glucose. Incubation with 100 nM rhodamine B or 175 nM DiOC_6_(3) were conducted for 20 min in the dark at 28 °C. After incubation, cells were harvested and resuspended in fresh HEPES buffer. The fluorescence was measured using a TECAN Infinite 200 microplate reader (Tecan Group Ltd., Männedorf, Switzerland) at λ_ex_ = 555 nm and λ_em_ = 579 nm for rhodamine B or at λ_ex_ = 476 nm and λ_em_ = 501 nm for DiOC_6_(3). Mitochondrial network was also visualized using fluorescence microscopy at appropriate wavelengths. The microscopic images, which present typical results from of the duplicate experiment, were captured with the Olympus BX-51 microscope (Olympus, Tokyo, Japan) equipped with the DP-72 digital camera and cellSens Dimension v1.0 software.

### 4.6. Determination of Cell Growth

Growth of yeast cells was analyzed on the liquid medium. Yeast cultures were cultivated for 24 h in a shaking incubator Titramax 1000 (Heidolph Insruments GmbH & CO. KG, Schwabach, Germany) at 1200 rpm at 28 °C. The growth was monitored turbidimetrically at λ = 600 nm using an Anthos 2010 type 17, 550 microplate reader (Anthos Labtec Instruments, Salzburg, Austria). Measurements were performed at 1 h intervals for 12 h and after 24 h of cultivation. The results are presented as a percent of changes in growth rate in comparison to control (cells of WT strain growing with 2% glucose concentration). The growth rate was calculated from exponential phase of growth using an appropriate formula [75].

### 4.7. Assessment of Cell Metabolic Activity

Relative metabolic activity of yeast cell, which can be treated as an equivalent of cell vitality, was determined with FUN^®^ 1 according to the manufacturer’s protocol (Molecular Probes) with modification described by Reference [76]. Incubation with 0.5 μM FUN-1 was conducted for 15 min in the dark at 28 °C. The fluorescence of the cell suspension was measured using a TECAN Infinite 200 microplate reader (Tecan Group Ltd., Männedorf, Switzerland) at λ_ex_ = 480 nm and λ_em_ = 500–650 nm. The metabolic activity of cells was expressed as a percent of changes the ratio of red (λ = 575 nm) to green (λ = 535 nm) fluorescence in comparison to control (cells of WT strain growing with 2% glucose concentration).

### 4.8. Estimation of Cell Size

The mean value of the cell size in the population was estimated through analysis of microscopic images captured with an Olympus BX-51 microscope equipped with the DP-72 digital camera (Olympus, Tokyo, Japan). Diameter of the cell was measured using cellSens Dimension software. Cell diameter was measured in two perpendicular planes for each cell and the mean value was used for calculations. For each yeast strain cultured in medium with different glucose concentrations, at least 300 cells were counted.

### 4.9. Determination of Yeast Cell Dry Weight

Cell biomass was determined by analysis of cell dry weight using moisture analyzer. Samples of the cell suspensions were taken from early exponential phase culture. Cells were centrifuged, washed 2 times in sterile Milli Q water, and suspended to density of 2 × 10^9^ cells/mL in sterile Milli Q water. Cell dry weight were determined by drying samples at 105 °C in MAC 50/NH Radwag moisture analyzer (Radwag, Radom, Poland). The drying program was set to end when no change in weight (lasting about 60s) was achieved. The data were expressed as a mg of cell dry weight per total amount of cells, but cell dry mass per single cell was also calculated.

### 4.10. Preparation of Cell Extracts

For the extraction, 5 × 10^8^ yeast cells from the exponential phase culture were used. Cells were centrifuged, washed twice with cold sterile water, and suspended in a 20 mM phosphate buffer with pH 6.8 containing 1 mM EDTA and 1 mM PMSF. The cells were disrupted with 0.5 mm glass beads in 7 cycles of 30 s with intervals for cooling the sample in ice and centrifuged. The supernatants were used for determination of protein and glucose-6-phosphate content in the yeast cell.

### 4.11. Determination of Protein Content in the Yeast Cell

Protein concentration was determined using the Bradford method. The absorbance of samples was measured after 10 min of incubation with Coomassie Protein Assay Reagent at room temperature using a TECAN Infinite 200 microplate reader (Tecan Group Ltd., Männedorf, Switzerland) at λ = 595 nm. The data were expressed as a mg per ml, but protein content per single cell was also calculated.

### 4.12. Determination of Glucose-6-Phosphate Content in the Yeast Cell

The level of glucose-6-phosphate in yeast cells was determined with Glucose-6-Phosphate Assay according to the manufacturer’s protocol (Sigma-Aldrich) with our own modifications. Previously prepared cell extracts were deproteinized with a 10 kDa MWCO spin Pierce Concentrator according to the manufacturer’s protocol (Thermo Scientific, Waltham, MA, USA). Deproteinized supernatants in a final volume of 50 µL were mixed with proper assay reagents supplied in the manufacturer’s kit. The absorbance of samples, proportional to the amount of glucose-6-phosphate, was measured after 30 min of incubation at room temperature using a TECAN Infinite 200 microplate reader (Tecan Group Ltd., Männedorf, Switzerland) at λ = 450 nm. The value of the blank was subtracted each time. The amount of glucose-6-phosphate in the samples was determined from the standard curve and data were expressed as pmol per µL.

### 4.13. Determination of NADPH, NADP^+^ Content, and NADP(H) Pool

NADP^+^, NADPH, contents in yeast cells were assessed with NADP/NADPH-Glo Assay according to the manufacturer’s protocols (Promega). Cells from early exponential phase culture were centrifuged, washed, suspended to density of 2 × 10^6^ cells/mL in PBS buffer, and used for determination purposes. Luminescence was recorded for 3 h using TECAN Infinite 200 microplate reader (Tecan Group Ltd., Männedorf, Switzerland). The value of the blank was subtracted each time. The results were presented as NADP^+^/NADPH ratio, which showed the share of NADPH and NADP^+^ in the total NADP(H) pool.

### 4.14. Pentose Phosphate Pathway Enzyme Activity Assays

The total dehydrogenase activity (understood as the sum of both glucose-6-phosphate dehydrogenase (G6PD) and 6-phosphogluconate dehydrogenase (6-PGD) activities) and separately the 6-phosphogluconate dehydrogenase activity were determined spectrophotometrically by measuring the rate of NADP^+^ reduction at 340 nm according to Reference [77], with our own modifications. Glucose-6-phosphate dehydrogenase activity was calculated by subtracting the activity of 6-phosphogluconate dehydrogenase from the total enzyme activity. To obtain the total dehydrogenase activity, 0.2 mM NADP+, 0.4 mM D-glucose-6-phosphate, and 0.4 mM 6-phosphogluconate as reaction substrates were used. The substrates were added to 100 mM Tris-HCl buffer with pH 8.0 containing 1 mM MgCl_2_. Addition of 5 µl cell extract (2 mg/mL) initiated the reaction. In turn, to obtain 6-phosphogluconate dehydrogenase activity only 0.2 mM NADP+ and 0.4 mM 6-phosphogluconate were used as reaction substrates. The kinetics of absorbance increase was recorded for 3 min using TECAN Infinite 200 microplate reader (Tecan Group Ltd., Männedorf, Switzerland) at λ = 340 nm. The data were expressed in arbitrary units.

### 4.15. Determination of Tryptophan and Riboflavin Contents

Tryptophan and riboflavin contents were estimated in the cell extracts using TECAN Infinite 200 microplate reader (Tecan Group Ltd., Männedorf, Switzerland) at the characteristic wave-lengths of λ_ex_ = 290 nm and λ_em_ = 325 nm; λ_ex_ =460 nm and λ_em_ = 535 nm, respectively, according to Reference [40]. The values were expressed in arbitrary units.

### 4.16. Proteasomal Activity Assays

The proteasome activity was determined with fluorogenic peptides Z-ARR-AMC (trypsin-like activity); Suc-LLVY-AMC (chymotrypsin-like activity); and Z-LLE-AMC (caspase-like activity). Briefly, cells were centrifuged, washed twice with cold sterile water, and suspended in protein extraction buffer (50 mM HEPES buffer with pH 7.8, containing 10 mM NaCl, 1.5 mM MgCl_2_, 1 mM EDTA, 1 mM EGTA, 250 mM sucrose, and 5 mM DTT). The cells were disrupted with 0.5 mm glass beads in 7 cycles of 30 s with intervals for cooling the sample in ice and centrifuged (14,000 *g*, 15 min, 4 °C). The degradation of fluorogenic peptide was measured by continuously monitoring the fluorescence of the reaction product, free 7-amino-4-methylcoumarin (AMC). The assay was performed in a protein extraction buffer supplemented with 2 mM ATP. The rate of fluorescence increase was measured using a TECAN Infinite 200 microplate reader (Tecan Group Ltd., Männedorf, Switzerland) at 37 °C for 60 min at λ_ex_ = 350 nm and λ_em_ = 440 nm. The values were expressed in arbitrary units.

### 4.17. Statistical Analysis

The results are presented as mean ± SD from at least three independent experiments (apart from determination of yeast reproductive potential). The statistical analysis was performed using the STATISTICA 10.0 software. The statistical significance of the differences between two yeast strains compared (the wild-type strain and mutant strain) were evaluated using the t-test for independent samples. The statistical significance of the differences between means of the three media compared was evaluated using one-way ANOVA with the Tukey post-hoc test. Homogeneity of variance was checked using Levene’s test. The values were considered significant at *p* < 0.05. Used designation: differences between strains * *p* < 0.05, ** *p* < 0.01, *** *p* < 0.001; differences between media a—different to medium with 0.5% glucose, b—different to medium with 2% glucose, c—different to medium with 4% glucose.

## 5. Conclusions

Deletion of the *HXK2* gene generates far-reaching metabolic consequences triggered by flux changes in interrelated intracellular metabolic pathways. These changes connected with fluxes in CCM pathways resulting in low overall biosynthetic capabilities and improved proteostasis seem to be the reason for higher reproductive potential observed in the Δ*hxk2* strain. This explains why increased respiration is observed but also shows that this is not a prerequisite for reproductive potential extension. Therefore, increased respiration seems to be an accompanying effect of the positive role of CR. Moreover, our study suggests that the high level of ATP observed in the Δ*hxk2* strain is not simply the result of increased respiration. It might be said that cells lacking Hxk2p get stuck in a specific “metabolic loop”. Deficiency of Hxk2p redirects glucose-utilization flux through respiration due to loss of the glucose repression signal and increased degradation of Mig1p but also due to lower glucose uptake, low glycolytic flux, and lower activity of the cAMP/PKA pathway. Redirection in the glucose-utilization pathway entails a decrease in cell macromolecular biosynthesis, which results in reduction of the cell size, cell dry weight, and protein content but also reduces cellular energetic costs and increases the level of recycling of cellular components by increasing proteasomal degradation. On the other hand, a high rate of respiration and increased ATP content requires higher level of ribose-5-phosphate production in the PP pathway for the de novo nucleotide synthesis, although saturated mitochondrial ATP production capacity may result in accumulation of glycolytic intermediates, which in turn play an important role in controlling the metabolic flux.

## Supplementary Materials

Supplementary materials can be found at https://www.mdpi.com/1422-0067/21/19/7313/s1.

## Abbreviations

CCM	Central Carbon Metabolism
MMP	Mitochondrial Membrane Potential
CR	Calorie restriction
CE	Calorie excess
