# Supplementary material for: Reproductive Potential of Yeast Cells Depends on Overall Action of Interconnected Changes in Central Carbon Metabolism, Cellular Biosynthetic Capacity, and Proteostasis
Source: Int J Mol Sci. 2020 Oct 3;21(19):7313. doi: 10.3390/ijms21197313 (PMC7582853; doi:10.3390/ijms21197313)
Supplement: Supplementary file 1 [file ijms-21-07313-s001.pdf]

Supplementary Materials

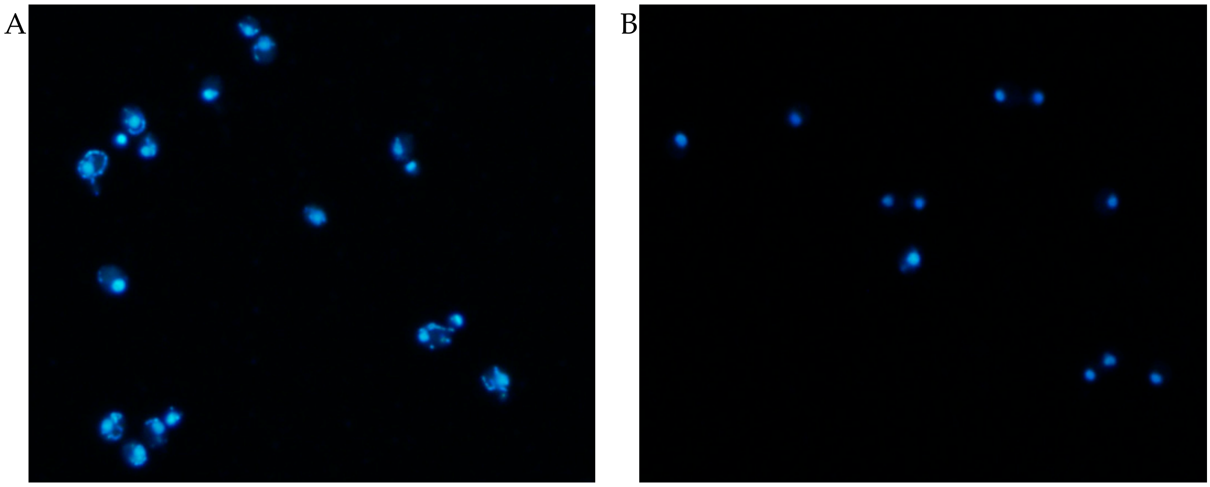

Fig. S1. The respiratory-competent (A) and respiratory-deficient yeast cells (B) stained with DAPI. To obtain respiratory-deficient cells, respiratory-competent cells of Δ*hxk2* yeast strain were treated with ethidium bromide according to the procedure described by Slonimski et al. (1968).

References:

Slonimski P.P.; Perrodin G.; Croft J.H., Ethidium bromide induced mutation of yeast mitochondria: complete transformation of cells into respiratory deficient non-chromosomal 'petites'. *Biochem Bioph Res Co* **1968**, 30:232-239.
